# Feasibility and safety study of a high resolution wide field-of-view scanning endoscope for circumferential intraluminal intestinal imaging

**DOI:** 10.1038/s41598-021-82962-2

**Published:** 2021-02-11

**Authors:** Lily L. Lai, Marta Invernizzi, Michael White, Chao Han, Huangfu Jiangtao, Helen Lu, Changhuei Yang, James Lin

**Affiliations:** 1 Department of Surgery, City of Hope, 1500 East Duarte Road, Duarte, CA 91010 USA; 2 Department of Electrical Engineering, California Institute of Technology, Pasadena, CA USA; 3 Department of Gastroenterology, City of Hope, Duarte, CA USA

**Keywords:** Cancer, Cancer imaging, Cancer screening, Gastrointestinal cancer

## Abstract

Global anal cancer incidence is increasing. High resolution anoscopy (HRA) currently screens for anal cancer, although the definitive test remains unknown. To improve on intraluminal imaging of the anal canal, we conducted a first-in-human study to determine feasibility and safety of a high-resolution, wide field-of-view scanning endoscope. Fourteen patients, under an IRB-approved clinical study, underwent exam under anesthesia, HRA, and imaging with the experimental device. HRA findings were photographed using an in-line camera attached to the colposcope and compared with the scanning endoscope images. Patients were followed up within 2 weeks of the procedure. The imaging device is inserted into the anal canal and the intraluminal surface is digitally photographed in 10 s and uploaded to a computer monitor for review. Ten patients completed imaging with the device. Three patients were not imaged due to severe anal stenosis. One patient was not imaged due to technical device malfunction. The device images were compared to the HRA images. No adverse event attributable to the device was reported. The intraluminal scanning endoscope can be used for circumferential anal canal imaging and is safe for clinical use. Future clinical studies are needed to evaluate the performance of this device.

## Introduction

Along with the other HPV-related malignancies such as cervical and oropharyngeal cancers, the overall incidence rate of anal canal cancer, particularly squamous cell carcinoma of the anus (SCCA), has been increasing worldwide over the past 30 years^1–5^. The oncogenic subtypes of human papillomavirus (HPV), most notably strains 16 and 18, are etiologically linked to SCCA^6,7^. The identification of HPV as a major contributing factor to the development of SCCA suggests that incidence patterns as well as demographic characteristics of the disease may have changed over time. Indeed, in a recent study, we demonstrated that the dramatic increase in annual percentage change after 1997 was associated with demographic changes consistent with the epidemiology of HPV transmission in the United States^8^. In addition, patients with a history of HPV-related cancers are at much higher risk of developing a second HPV-related cancer^9^.

Screening for HPV-related cancers such as anal cancer leads to early detection and less radical treatments to cure the disease. At present, there remains no definitive method to screen for anal cancer although the use of a colposcope through a clear plastic anoscope to visualize the luminal surface of the anal canal, known as high resolution anoscopy (HRA), has been widely described. To improve on this, we designed and built a prototype device to image the anal canal^10^. The device relies on widely available commercial card scanning technology repurposed for clinical use. In this report, we detail our first-in-human clinical testing of the feasibility and safety of imaging the anal canal using this device.

## Methods

### Device development

The design, development, build, and initial testing of the device in an animal model have been reported^10^. In brief, the imaging device was designed and built to circumferentially image the lumen of the anal canal using the imaging principles adopted from those of commercial flatbed and business card scanners. The adoption of such technology for our clinical application capitalizes on the economy-of-scale optimizations already in place for such scanners.

The light source consists of three RGB LED emitters coupled to a light guide that is carefully designed to provide uniform lighting along its entire length. As the sample of interest is illuminated, the reflections are collected by a SELFOC lens array (SLA) and projected onto the linear image sensor. The SLA consists of a linear collection of cylindrical gradient-index lens (GRIN lens). These arrays are highly compact optical systems that are able to optically image a thin strip of the sample’s surface onto a linear sensor array in a 1:1 unmagnified fashion with resolution on the order of tens of microns. In combination with linear translation, the document scanners can cover a large area while maintaining the fidelity of the image quality. In effect, the system collects one line’s worth of image information. By scanning across the sample, a 2D image is generated from multiple lines of images.

The imaging module from a palm-size business card scanner (Opticard 821, Plustek, Inc., Priorslee, Telford, UK) was removed. All the components of the imaging module were then realigned and mounted onto an axis by a customized 3D-printed adaptor. The axis was connected to a stepper motor (28BYJ-48, Kootek) with gears. The mounted imaging module was fixed inside a glass tissue culture tube of diameter 38 mm (Bellco Glass, Vineland, NJ). In effect, the business card scanner was adapted to perform cylindrical scans.

**Figure 1:**
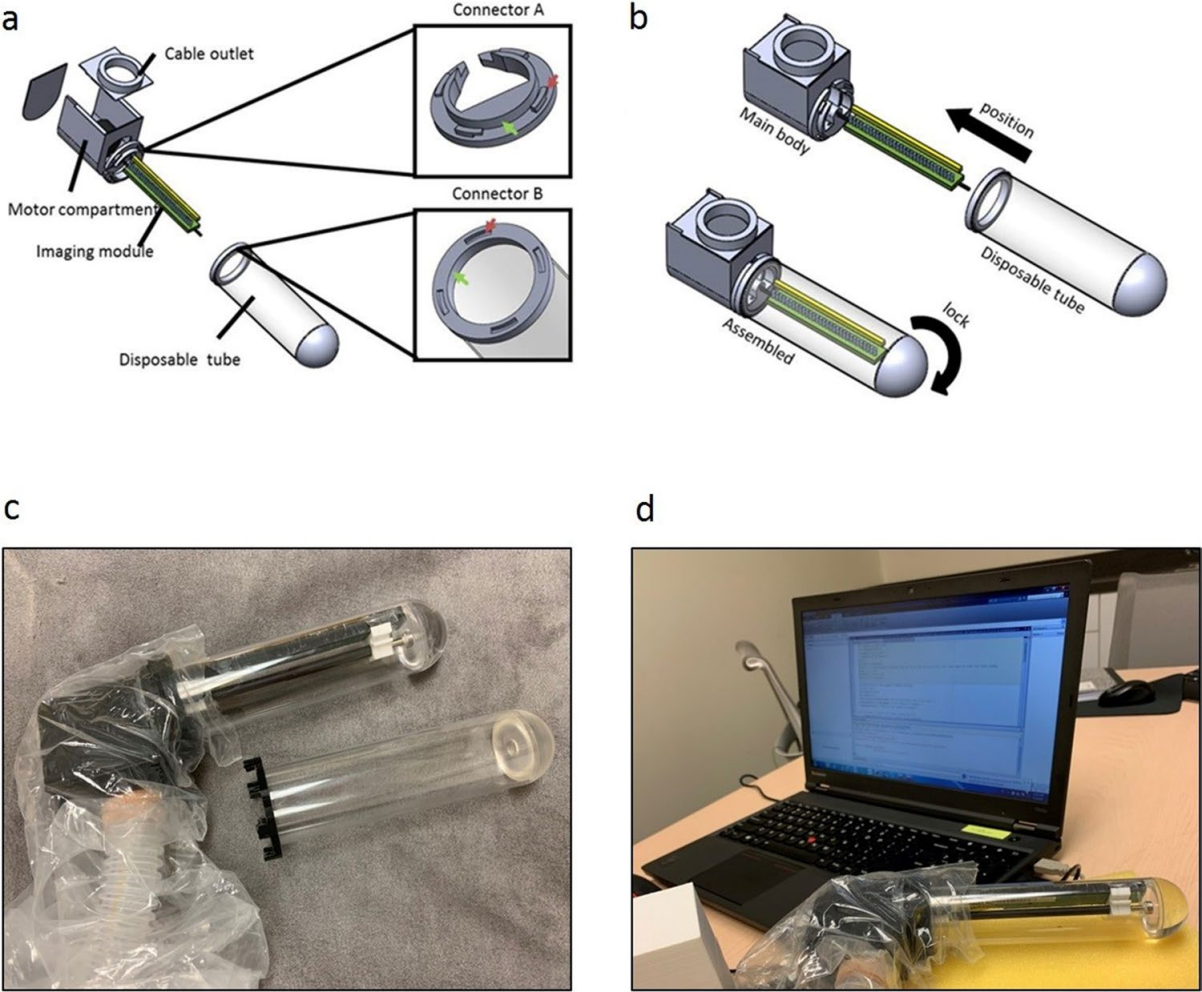
The patient centered re-design of the imaging device. (**a**) The main body consists of the motor compartment and the imaging module. The disposable medical-grade clear polycarbonate tube is mounted and secured to the main body by the use of two built-in connectors. Connector A on the main body and Connector B on the tube are as shown, respectively. (**b**) The tube mounting process for the new AnCam requires insertion of the imaging module into the transparent tube, turning the tube and the main body to engage Connectors A and B. The patient-facing tube is single use and disposable. The non-patient facing main body and tubing are covered with a single use plastic disposable sheath. (**c**,**d**) Photographs of the scanning endoscope system used in the clinical study.

To obtain the image of the intraluminal cylindrical surface of the anal canal, we incorporated the imaging module into an optical quality transparent tube selected to approximate the size and shape of currently used anoscopes. The anoscope diameters range from 15 to 30 mm, with the most common outpatient anoscope sized at a diameter of 20 mm. To image the anal canal epithelium in one scan, we used a tube with a diameter of 38 mm to stretch the anal folds ensuring that the entire anal canal epithelium is fully apposed to the tube surface. The components of the imaging module were individually realigned to fit the geometry of the tube and to ensure the sample on the outer surface of the tube could be imaged correctly onto the linear image sensor (Fig. 1).

By the rotational scan of the imaging module inside the tube at a constant speed, the entire circumferential image of the cylindrical lumen can be obtained. The device rapidly scans and records the intraluminal 360° image in 10 s. Image processing results in a cropped, linearized image available on the computer monitor in seconds for clinical review. The device captures the whole image of the anal canal with a resolution of 89 μm, a maximum FOV of 100 mm × 120 mm, and a depth-of-field (DOF) of 0.65 mm at 5.9 line pairs per mm (lp/mm).

The device was tested in a porcine model to determine ability to image the lumen of the anal canal. The processed image was cropped, linearized, and digitally zoomed in and was compared to HRA images taken at the same time. Reproducible serial images, taken after successive removals and reinsertions of the device in the porcine model, were also demonstrated, as have been previously reported^10^.

### Patient-centered design

The device was redesigned to incorporate patient-centered principles for clinical use. The patient facing components such as the cylindrical portion of the probe are easily removed and disposed by designing and building two separate and detachable components: the main body and the disposable tube as shown in Fig. 1. The main body houses the motor and cables. In addition, an outlet for cables packaged in medical tubing protects the instrument and cables from contamination (Fig. 1a). The transparent disposable tube is fabricated from optically transparent medical grade polycarbonate and covers the scanner (Fig. 1a). The quick connection between the main body and the tube are facilitated by two connectors. The surfaces of the Connector A and Connector B (green and red arrows in Fig. 1a) are designed to lock into position with a simple rotation (Fig. 1b). Disposable and sterile ultrasound plastic sheaths are used to protect the handle and the cable housing (Fig. 1c). The clear polycarbonate tubes and plastic sheaths are discarded after each patient use. The handle, housing, and computer are cleaned using cleaning solutions approved by the institution and as per standard operating room protocol. The entire system is shown in Fig. 1d.

### Clinical study

The clinical study underwent City of Hope Institutional Research Board approval and oversight. The clinical trial was conducted in accordance with relevant guidelines and regulations. Informed consent was obtained from all patients recruited to the clinical study. Once consented, the patients were then enrolled and were scheduled for anal cancer screening or surveillance.

In the operating room, careful digital rectal exam and initial anoscopy and colposcopy without acetic acid (pre-acetic acid HRA) was completed. Pre-acetic acid images were taken using the in-line camera attached to the colposcope. After the pre-acetic HRA examination was completed, the patients underwent anal canal imaging using the investigational imaging device. The device was inserted into the anal canal in the same manner as an anoscope. Pre-acetic acid images were taken by the device. The device was withdrawn. Acetic acid-soaked gauze was inserted into the anal canal for 5 min. The gauze was removed. The device was reinserted into the anal canal. Post-acetic acid images were taken. At the completion of the images, the device was withdrawn. Acetic acid was again applied. Post-acetic acid HRA was completed. Biopsies, if deemed necessary on HRA, were obtained per standard HRA techniques. Patients were called or seen in clinic within 2 weeks after the procedure as follow-up. Adverse events were recorded and attributed per institutional protocol.

## Results

### Demographics

Table 1 lists the demographic and clinical characteristics of the patients. The patients were recruited to participate in this study because they were scheduled for surveillance exams given their history of HPV-related cancers and neoplasms. Of the 14 patients, the patients had a total of 15 HPV-related diagnoses. The diagnoses included anal cancer (n = 8), anal intraepithelial neoplasia (AIN) (n = 4), cervical cancer (n = 2), and anal condyloma (n = 1). One patient had two cancers, anal and cervical cancer. The anal cancer patients were diagnosed and treated with chemoradiation within a mean of 4.6 years (range 1–10 years) of enrollment in this study. There were 8 females and 6 males. The mean age of the patients was 61 years (range 47–72 years). The majority of the patients had abnormal findings on HRA (10 of 14, 71%) which were identifed and biopsied during the procedure. Of these, AIN was confirmed in 3 patients. The other specimens were noted to have HPV-related changes (n = 2), chronic inflammation (n = 1), or normal epithelium (n = 4).

**Table 1:** Demographic and clinical data of patients.

Patient	Age	Sex	Clinical indication for surveillance	HRA findings	Histopathology results
1	54	F	Cervical dysplasia/anal cancer	Acetowhite area	AIN2
2	56	F	Anal cancer	Acetowhite area, anal condyloma, fibrotic area	Mild AIN1 with koilocytosis and papillary architecture
3	68	M	AIN	Acetowhite area	Benign squamous epithelium with lentigo simplex
4	68	M	Anal cancer	None	No biopsy
5	70	M	AIN3	None	No biopsy
6	63	F	Anal cancer	Fibrotic lesion	No biopsy
7	70	M	Anal cancer	None	No biopsy
8	65	F	Anal cancer	Acetowhite area	Squamous mucosa with chronic inflammation; no evidence of dysplasia or malignancy
9	72	F	Cervical cancer/anal cancer	Acetowhite area	No diagnostic abnormality
10	56	F	Cervical cancer	Acetowhite area	Anal papillae with focal HPV changes, no squamous dysplasia
11	57	F	AIN 3	Acetowhite area, anal condyloma	No dysplasia or carcinoma; anal condyloma
12	56	F	Anal cancer	Fibrotic lesion	No evidence of malignancy or dysplasia
13	47	M	HPV anal condyloma	Acetowhite area, anal condyloma	Colonic mucosa with no diagnostic abnormalities; condyloma acuminatum with koilocytosis negative for high-grade dysplasia or carcinoma
14	53	M	AIN 3	Acetowhite area; palpable mass	AIN3 and marked chronic inflammation

### Feasibility

Of the 14 patients, 10 patients underwent successful imaging of the anal canal with the device. Of the four failures, three were due to the inability to pass the device into the anal canal. These patients had anal stenosis secondary to previous anal cancer treatment with chemoradiation. There was a technical device malfunction in one patient in which the device failed to record images at the time of the procedure.

### Images

**Figure 2:**
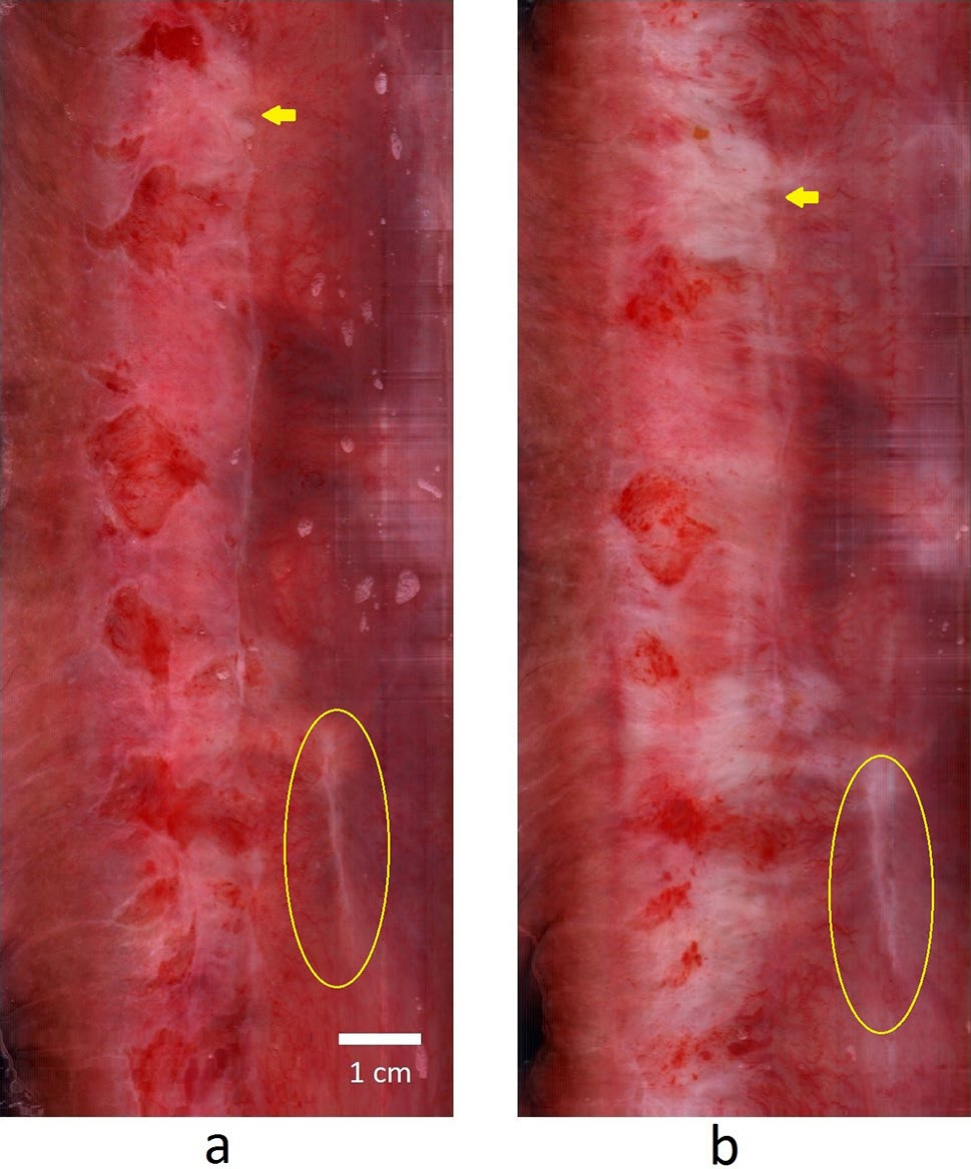
Serial images demonstrate acetic acid induced changes in the anal canal epithelium. The wide field-of-view (FOV) image of the anal canal (**a**) pre-acetic acid and (**b**) post-acetic acid. The increased whiteness (arrow) is the noted effect of acetic acid instillation. The yellow circle identifies a surgical scar in the pre-acetic acid and in the post-acetic acid images.

**Figure 3:**
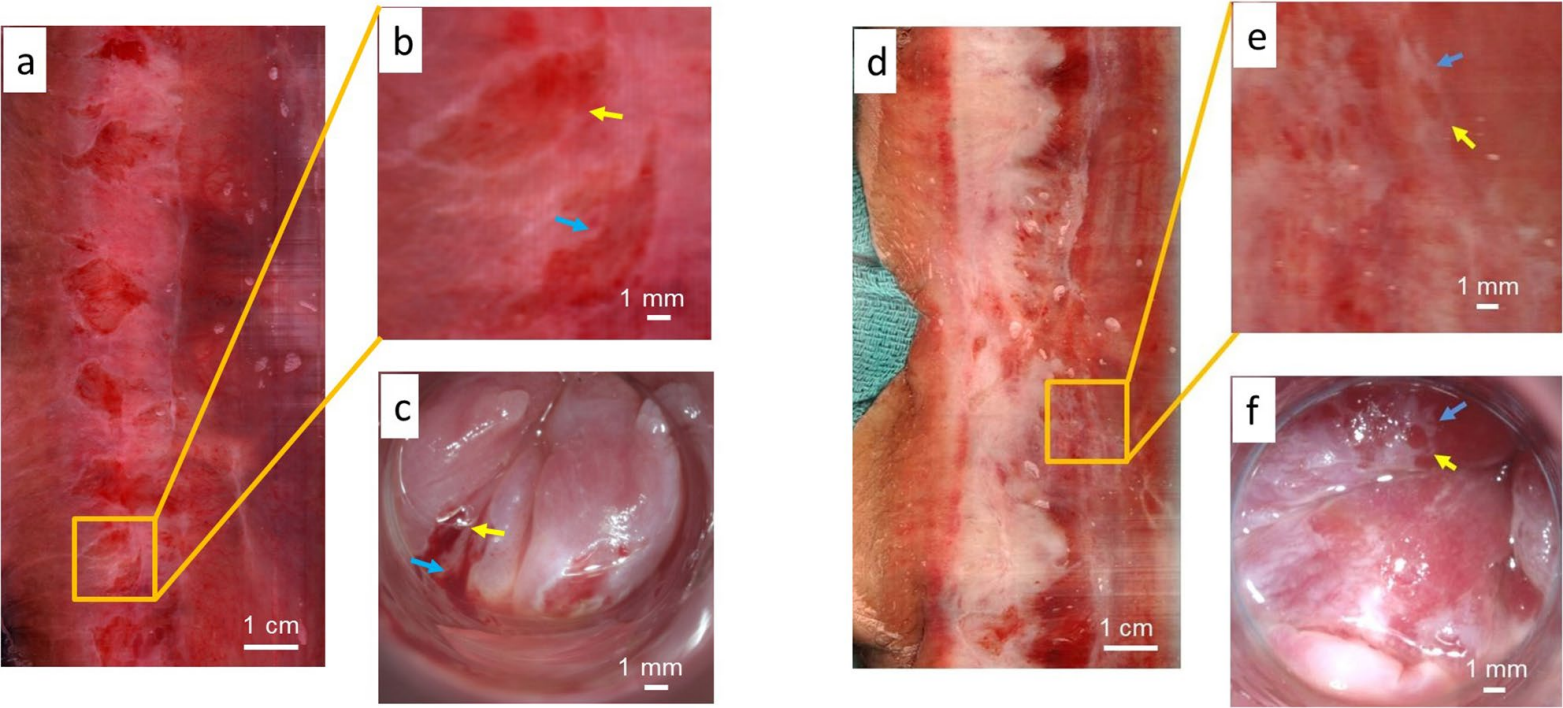
Wide FOV circumferential images are comparable to HRA images of the anal canal. Two examples of anal canal imaging are shown. (**a**,**d**) The wide FOV circumferential image of the entire anal canal. (**b**,**e**) A digital zoomed-in image of an area of interest. (**c**,**f**) The colposcope (HRA) image of the same area shown in (**b**) and (**e**). The colored arrows point to the same areas of interest as imaged using the device and as imaged through HRA.

Each wide FOV intraluminal circumferential image was completed in 10 s. Data processing of the raw digital image resulted in a cropped, linearized image that was uploaded to the monitor within 20 s for physician review. Serial images were taken before (Fig. 2a) and after acetic acid (Fig. 2b) was applied. The device images (Fig. 3b,e) were qualitatively compared with HRA images (Fig. 3c,f).

### Safety

The patients were followed up either in clinic or with a phone call within 2 weeks of the procedure. There was no adverse event attributed to the scanning endoscope. Five patients had postoperative bleeding (Grade 1) without need for intervention and 4 patients reported rectal pain (Grade 1) treated with pain medications. There were no Grade 3–5 adverse event. Table 2 lists the reported adverse events, grade, and attributions.

**Table 2:** Adverse events and attributions.

Adverse event^a^	Total N	Grade 1	Grade 2	Grade 3	Grade 4	Grade 5	Attributed to device
Hypertension	3	2	1	0	0	0	No
Rectal hemorrhage	5	5	0	0	0	0	No
Rectal pain	4	3	1	0	0	0	No
Abdominal pain	1	1	0	0	0	0	No
Diarrhea	1	1	0	0	0	0	No

^a^CTCAE Version 5.0, published: November 27, 2017, US Department of Health and Human Services, National Institutes of Health, National Cancer Institute.

## Discussion

Over the last three decades in the United States, the incidence of anal cancer has risen three-fold with an annual rate of increase of 7%^8^. In patients with advanced disease, survival is less than 45% at 5 years and requires highly morbid treatments including chemotherapy, chemoradiation, and radical resection with permanent colostomies^11^. However, when detected early through screening, anal cancer can be cured with local resection and/or ablative techniques^12^.

HPV is associated with 65–89% of all SCCA and is implicated as a cause of the disease^13,14^. The epithelial changes in the anal canal associated with anal HPV infection have been well described by Palefsky et al.^15^. Their pioneering work in the field established the use of high resolution anoscopy (HRA) and HRA-directed biopsies as the current standard in screening and diagnosis of precursors of anal cancer [high grade squamous epithelial lesion (HSIL)] and early anal cancer [superficially invasive squamous cell carcinoma (SISCCA)]^16^. Screening at-risk patients has resulted in stage shifts in SCCA, with more frequent detection of malignancy at earlier stages^17^.

HRA screening, considered the gold standard, is both a screening and diagnostic test. To achieve detection of HSIL and SISCCA, the test requires multiple and serial biopsies. Between 60 and 80% of patients are biopsied at their first HRA^18,19^. About 47% of high-risk patients who undergo biopsies are found to have normal histology in the specimens^18,20,21^, underscoring the modest correlation between clinical impressions that guided the biopsy with the histopathologic results (kappa statistic of 0.36)^22^. HRA sensitivity and specificity improve with serial biopsies^18^, although inter- and intra-observer reliability of HRA have yet to be fully characterized and reported. Anal cytology and anal HPV testing have been proposed to identify patients most likely to benefit from HRA screening. However, correlation between cytology results and HPV status with anal cancer remains to be fully tested^19,20,22,23^. Although HRA is the current gold standard for anal cancer screening, the procedure averages over 15–30 min; requires multiple steps in the set up and execution; depends on the skill set of the provider to identify abnormal areas to biopsy; and demands a high level of histopathologic resources for support. In addition, the potential for pain and discomfort with serial HRA exams and requisite biopsies further decreases likelihood of widespread acceptance of HRA as a screening modality. A triage test that allows for serial and longitudinal follow-up without the need for serial tissue biopsies is critical to improve effective screening for anal cancer. Whether screening, with or without subsequent treatment, decreases incidence and mortality of SCCA is actively being studied in ongoing prospective clinical trials.

However, limited data does suggest that early detection is important. A recent retrospective study of patients at risk for anal cancer who undergo anal cancer surveillance and those who did not demonstrated that anal cancers that developed during surveillance were earlier stage and required less aggressive intervention to treat^24^.

We have designed and developed a novel device able to complete circumferential wide FOV scanning of the anal canal with digital high-resolution images in less than 10 s. In this study, we demonstrate the feasibility of our first-in-human use of the device. Intraluminal images of patients were completed and are qualitatively comparable to images taken at the time of HRA, as shown in Figs. 2 and 3. Overall, the resolution of the HRA images at high magnification is better than the images taken with the scanning endoscope which is limited by pixel density [600 pixels per inch (ppi)] of the image sensor. Future development will improve on the scanning endoscope image resolution by incorporating a higher ppi image sensor.

Possible advantages of this device for anal cancer screening include the simplicity of its use and the decreased length of time in contrast to current HRA screening. HRA requires setup of both an anoscope and a colposcope. The current device reduces the multiple HRA steps into one step and completes wide FOV intraluminal circumferential imaging in 10 s. This device has the potential to change screening from a 15–30-min uncomfortable procedure to a 1-min completed test. The marked reduction in time for a screening test has the potential to increase efficiency and availability of screening to at-risk patients.

In addition, the digital images are reproducible and serial images can be compared. The scanning of the anal canal is set to begin at the anterior location of the anal canal and scans in a clockwise direction. The image is then linearized. The top and bottom of the linearized image are anterior; the middle of the linearized image is posterior. Serial images can be qualitatively compared as shown in Fig. 2 in which the scanning endoscope was inserted before addition of acetic acid (Fig. 2a) and after addition of acetic acid (Fig. 2b). Anal mucosal findings (e.g. as shown encircled with the yellow line) are qualitatively comparable between images. In addition, with initiation of the clockwise scan at the anterior location of the anal canal in a patient in lithotomy position, the scar (yellow circle) seen in Fig. 2 is localized at the right anterolateral anal canal. The ability to localize enables directed biopsies. Although the anal mucosal changes highlighted with Lugol’s solution were not imaged in this study, we anticipate that images taken before and after Lugol’s solution, as with acetic acid, application, can be completed using the scanning endoscope to improve on the detection of anal neoplasm. Two of the 14 patients had repeat imaging with the device within a 6-month interval (data not shown), arguing for the utility of the imaging device in serial follow-up. This paradigm of a screening test as triage to further workup is more consistent with established cancer screening programs such as breast cancer, for which screening mammographic abnormalities trigger callbacks for further diagnostic workup.

Finally, the images obtained from this device are digital and transmissible. Review of the digital data can be done remotely and not necessarily in real time, much like imaging for breast cancer screening. The scanned images can be reviewed and read by multiple clinicians, improving on the accuracy and consistency of determination of abnormal areas. In this study, we document the feasibility of the use of this imaging device. Additional studies are required to better evaluate the performance of the device in detecting anal dysplasia and cancer when compared with HRA.

We were unable to use the scanning endoscope to image four of the 14 patients enrolled into this study. The current circumference of the disposable tube precluded insertion into three patients with anal stenosis, an occasional sequela of chemoradiation used to treat anal cancer. In addition, the size of the current scanning endoscope may require downsizing since superficial, epithelial injuries can be seen in the images. We have begun to redesign the device to include disposable tubes of varying sizes. Different tube sizes will allow for better patient accommodation while maintaining the principle of stretching the anal epithelium to decrease the likelihood of missed lesions hidden in anal mucosal folds. The fourth instance of inability to obtain images was the result of technical device malfunction at the time of the procedure. To circumvent such an issue in the future, a second system will be available as a backup.

In addition to feasibility, we also describe the safety of the imaging device for use in patients likely to need anal cancer screening or surveillance. Among the 14 patients studied, adverse events were few and of low grade. None of the reported adverse events were attributed to the device.

In summary, the potential advantages of a wide FOV scanning endoscope for intraluminal imaging of the anal canal imaging includes ease of use, speed, and acquisition and analysis of objective and reproducible digital images. In addition, the device platform may serve as a framework to incorporate more innovative optical technologies, such as light filters for multispectral imaging, to improve on the imaging capabilities of the current prototype. Data analysis of the serial digital images using deep neural networks may lead to automated diagnosis of anal canal dysplasia and cancer in the future and may obviate the need for biopsy that is the current standard of care. Potential applications of this device for imaging outside the anal canal include other cancer types such as melanoma and adenocarcinoma of the rectum. Finally, ongoing development may enable application for use in other disease sites such as the esophagus or vagina. Further study is needed to define use and to document benefit.
